# Foliar Nutrient Distribution Patterns in Sympatric Maple Species Reflect Contrasting Sensitivity to Excess Manganese

**DOI:** 10.1371/journal.pone.0157702

**Published:** 2016-07-08

**Authors:** Denise R. Fernando, Alan T. Marshall, Jonathan P. Lynch

**Affiliations:** 1 Department of Ecology, Environment and Evolution, La Trobe University, Bundoora, Victoria, Australia; 2 Analytical Electron Microscopy Laboratory, La Trobe University, Bundoora, Victoria, Australia; 3 Department of Plant Science, The Pennsylvania State University, University Park, Pennsylvania, United States of America; CINVESTAV-IPN, MEXICO

## Abstract

Sugar maple and red maple are closely-related co-occurring tree species significant to the North American forest biome. Plant abiotic stress effects including nutritional imbalance and manganese (Mn) toxicity are well documented within this system, and are implicated in enhanced susceptibility to biotic stresses such as insect attack. Both tree species are known to overaccumulate foliar manganese (Mn) when growing on unbuffered acidified soils, however, sugar maple is Mn-sensitive, while red maple is not. Currently there is no knowledge about the cellular sequestration of Mn and other nutrients in these two species. Here, electron-probe x-ray microanalysis was employed to examine cellular and sub-cellular deposition of excessively accumulated foliar Mn and other mineral nutrients *in vivo*. For both species, excess foliar Mn was deposited in symplastic cellular compartments. There were striking between-species differences in Mn, magnesium (Mg), sulphur (S) and calcium (Ca) distribution patterns. Unusually, Mn was highly co-localised with Mg in mesophyll cells of red maple only. The known sensitivity of sugar maple to excess Mn is likely linked to Mg deficiency in the leaf mesophyll. There was strong evidence that Mn toxicity in sugar maple is primarily a symplastic process. For each species, leaf-surface damage due to biotic stress including insect herbivory was compared between sites with acidified and non-acidified soils. Although it was greatest overall in red maple, there was no difference in biotic stress damage to red maple leaves between acidified and non-acidified soils. Sugar maple trees on buffered non-acidified soil were less damaged by biotic stress compared to those on unbuffered acidified soil, where they are also affected by Mn toxicity abiotic stress. This study concluded that foliar nutrient distribution in symplastic compartments is a determinant of Mn sensitivity, and that Mn stress hinders plant resistance to biotic stress.

## Introduction

The essential trace element manganese (Mn) is integral to photosynthesis, free radical mitigation and redox processes, yet certain conditions of soil chemistry, climate and/or genetic predisposition can render it toxic to plants [1]. The importance of Mn as a plant nutrient and its phytotoxic effects have featured in the scientific literature for at least two centuries, however there is a lack of consensus about the physiology of Mn stress [2–6]. Manganese is ubiquitous in soil, occurring primarily as three easily interchangeable oxides of Mn(II), Mn(III) and Mn(IV) [1], of which only Mn(II)-oxide is soluble and available for plant uptake via the xylem. Relatively minor shifts in soil chemistry and/or climatic conditions can enhance soil-Mn bioavailability to levels potentially deleterious to plants [7–14]. In the field, soil Mn concentrations coupled with seasonal variation in rainfall and temperature have a strong bearing on plant over-exposure to Mn(II), while genetics largely determines the uptake of and physiological response to high shoot-tissue Mn concentrations. Soil acidification and waterlogging are often key factors in geographic regions to which this problem is common [1]. The enhanced solubilisation of soil-Mn also triggers nutritional deficiencies since Mn(II) outcompetes similar ions such as Ca(II) and Mg(II) for plant uptake. Given that the manifestation and extent of Mn phytotoxicity as observed in the field largely hinges on climatic variables, stress symptoms commonly are seasonally heterogeneous.

Changing global weather patterns may harm healthy plant ecosystems when inherent balances between key abiotic and biotic factors become skewed [15–17]. Soil acidification brought upon by acid deposition has been identified in North American studies as likely contributing to the ill-health of its native forests [5, 7, 18–24]. These and other reports support the wider notion of a ‘cascade effect’ linking nutritional imbalance, metal toxicity, free radical damage, heightened susceptibility to external factors such as pest and pathogen attack, drought, overexposure to sunlight, atmospheric ozone etc. The detrimental interaction of sunlight and excessively accumulated foliar Mn manifest in photobleaching damage to chloroplasts and oxygen free-radical stress has been reported for common bean and sugar maple [9, 13], pointing to the possible broad-scale negative impacts of extended periods of sunlight exposure [15]. Soil heating and drying contributes to Mn toxicity, as does high rainfall via strongly-reducing hypoxic conditions induced by waterlogging [11, 12, 25]. Altered rainfall patterns and other climatic variables therefore could exacerbate the potency of interactive plant and soil processes that drive Mn toxicity.

Controlled experiments and field studies documenting foliar Mn overaccumulation in North American forest trees have also identified the notable susceptibility of sugar maple (*Acer saccharum* (Sapindaceae)) to Mn stress [20, 23, 26]. An evaluation of nine tree species common to the eastern forests of North America [23] found that only sugar maple had a negative growth response to Mn overexposure. While compositional shifts within this globally significant biome may be attributed to acid rain, logging, coal mining, land reclamation, etc., the likely contribution of these factors toward Mn phytotoxicity has drawn little attention. The geographic distribution of sugar maple overlaps that of its close relative, red maple (*A*. *rubrum*), both characteristic biome species, of which the latter is notably less susceptible to nutrient imbalance [13]. Foliar-Mn elevation in sugar maple stands on acidified host substrates in North America had been widely reported [19, 21, 27, 28] prior to description of certain key physiological processes of Mn toxicity stress in both sugar and red maple [5, 13, 22, 23, 26, 29]. Controlled experiments showed that Mn treatments under conditions of high light exposure resulted in limited leaf CO_2_ exchange rates and stomatal conductance. This occurred to a greater extent in sugar maple than in red maple [13], while field studies linked high foliar Mn to reduced carboxylation efficiency and photo-oxidative stress [22, 29]. Mycorrhizal studies [13] have further illuminated the susceptibility of sugar maple to nutrient imbalance on acid soils. As reviewed by St Clair et al. [13], it is well documented that in North America sugar maple is notably vulnerable to a range of associated abiotic and biotic stresses including insect damage. Poor foliar nutritional status has been implicated in reduced chemical-defence capacity and long-term decline in tree health.

Manganese stress in plants although extensively documented, is explained in the literature via two major divergent hypotheses based either on symplastic [9], or apoplastic [4, 30, 31] processes. The symplastic hypothesis proposes that Mn toxicity acts via photo-oxidative stress, a process exacerbated by light, temperature, and other environmental factors, whereas according to the apoplastic hypothesis Mn stress damage will not be sensitive to these climatic variables. This discord warrants evaluation in the light of predicted changes to climatic variables [15], which when factored into the symplastic hypothesis has potentially far-reaching implications for enhanced Mn phytotoxcity. It is plausible then that misinterpreting Mn phytotoxicity could result in failure to recognise broad patterns of incrementally rising Mn stress directly due to shifting climate.

The Allegheny Plateau in Pennsylvania is a region within the eastern North American forest biome recognised as experiencing nutritional stress [21]. While soil properties including history of glaciation, parent material, and slope position have a strong bearing on plant nutrition, the overriding effects of soil acidification via acid precipitation are clearly evident [19]. Variability in foliar-Mn concentrations according to slope position on the study site had previously been demonstrated, i.e. that trees on buffered substrates of the lower slopes remain relatively unaffected by soil acidification compared to those on sites upslope. Sugar and red maple occur across both habitats, as well as on glaciated and unglaciated landscapes; with those on weathered soils exhibiting consistently elevated foliar Mn concentrations, albeit with some seasonal variation. The disparity in their susceptibilities to nutritional imbalance as widely documented for upslope trees presents a useful case study for examining interaction between plant Mn overexposure and response to soil acidification.

The aims of this investigation were several-fold, i.e. 1) to investigate foliar Mn disposal in two closely-related plant species differentially affected by Mn overaccumulation, 2) to address the question as to whether Mn phytotoxicity is predominantly symplastic or apoplastic, and 3) test the hypothesis that disruption to the primary photosynthetic surfaces, i.e leaf adaxial surfaces, correlates with elevated foliar Mn levels and nutritional stress.

## Materials and Methods

### Field sampling of plant material

Red maple and sugar maple leaves were sampled in late summer when their green-leaf Mn concentrations peak. Two different slope locations, i.e. upslope and downslope, were selected at Hardwood Ridge (41° 42.566′ N, 77° 55.488′ W) on the Allegheny Plateau, Pennsylvania (USA); on the basis of existing knowledge about this site [22]. It is well established that on this site, unbuffered acidified soils upslope drive foliar Mn overaccumulation in several tree species including red and sugar maple, while trees on buffered soils downslope are unaffected by Mn. It is also well established that unlike red maple, sugar maple on acidified soil is affected by Mn toxicity. The sampling strategy adopted here was premised upon consistent findings of numerous published field and controlled studies describing Mn stress in sugar maple at this site and others [5, 13, 22, 23, 26, 29]. Two trees each of sugar maple and red maple were sampled at both slope positions for a range of analyses, and also for the lodgement of herbarium vouchers (S1 Table). The trees were numbered 1 to 8 with the following notation: 1, 2 sugar maple upslope (SM/U); 3, 4 sugar maple downslope (SM/D); 5, 6 red maple upslope (RM/U); 7, 8 red maple downslope (RM/D). Uppermost canopy leaves maximally exposed to sunlight were targeted because visible and UV radiation generate reactive oxygen species (ROS) that cause oxidative stress exacerbated by excess foliar Mn [13, 23] As detailed below, leaf material was sampled for the purposes of bulk-tissue chemical analysis, image analysis, light microscopy (LM), transmission electron microscopy (TEM), and scanning electron microscopy energy dispersive spectroscopy (SEM EDS). Herbarium vouchers were pressed in the field and lodged at the Pennsylvania Agricultural College Herbarium, The Pennsylvania State University.

### Leaf chemical analyses

Ten mature fully expanded leaves from each tree were pooled, dried and finely milled; from which ~0.5 g was digested in 5 ml heated concentrated (70%) nitric acid (HNO_3_). The digestate was diluted to 50 ml with deionised water, filtered and analysed by ICP-OES against a series of similarly acidified standards to yield leaf-tissue concentrations of boron (B), sodium (Na), Mg, aluminium (Al), phosphorus (P), S, potassium (K), Ca, Mn, iron (Fe), copper (Cu) and zinc (Zn).

### Image analysis for surface damage

Forty to seventy fresh leaves (per tree) were photographed from a fixed distance directly above, against a white background and a scale-bar. Leaf adaxial surface damage was quantified using ImageJ® (National Institutes of Health, Bethesda, Maryland, USA) software. Absent leaf margins were digitally estimated so as to enable quantification of missing leaf area against total leaf area. Localised surface discolouration was also quantified as a fraction of the total leaf area. For each leaf, missing tissue and localised discolouration percentages were pooled to obtain a quantitative estimate of overall surface-disruption. The effect of slope on leaf damage for each species was tested using arcsin-transformed data (IBM^®^ SPSS^®^ Statistics, Version 22).

### Light microscopy (LM)

Areas of healthy fresh leaf laminal tissue lacking surface damage/necrosis etc were sampled for microscopy. They were processed using a method modified from [32], i.e., by gluteraldehyde fixation in HEPES buffer, OsO_4_ post fixation, ethanol dehydration, resin infiltration and embedding in Spurrs^®^. Sections (0.5–1 μm) cut with a glass knife on a microtome (Leica UC6, Leica Microsystems, Deerfield, IL USA) were stained with toluidine blue, examined by light microscopy (Olympus BX51, Olympus America, Center Valley, PA USA), and photographed with a CCD camera (Jenoptik ProgRes CFscan, Rochester, NY USA).

### Scanning electron microscopy and x-ray microanalysis (SEM EDS)

Leaves sampled in the field were immediately wrapped in damp paper towel, sealed in plastic, and stored cool for processing in no later than 24 hrs, as determined by verified protocols [32–34]. Leaf lamina discs (1 cm) were punched out and immediately cryo-fixed by slam-freezing on a liquid-nitrogen (LN)-cooled metal mirror cryofixation device (Leica EM MM80, Leica Microsystems, Deerfield, IL USA), and stored in LN. Examination of cellular elemental contents in biological material requires initial cryo-fixation to immobilise metabolic processes, after which there are several proven methodological options for preparing and examining samples to obtain data that reliably represents cellular contents *in vivo* [34]. For this study, logistics and available options determined that the method used by Bidwell et al [35] modified from an original protocol by Marshall [36] was the most suitable. Numerous other studies including plant science research have previously successfully employed freeze-substitution sample preparation methodology for *in vivo* microbeam analysis [37–42]. Here, sample preparation was undertaken in a desiccated environment, and entailed slow freeze-substitution with a dry non-polar solvent mixture (10% acrolein in diethyl ether) over a two-week period commencing at LN temperature and gradually being elevated to room temperature. Substituted tissues were retained desiccated, infiltrated with and embedded in desiccated Spurrs^**®**^ resin, and stored desiccated.

For SEM EDS elemental analysis, each resin block was hand-trimmed, mounted on a SEM sub-stage and planed cross-sectionally using a glass knife on an ultramicrotome (Reichert Jung FC4E, Leica Microsystems) to reveal the leaf cross-sectional surface. Specimen electrical charging under the electron beam was mitigated by several steps, primarily by initially pre-coating the planed sample block with platinum (Pt) (10 Å) to cover the vertical sides, then replaning off the anaytical surface to remove Pt from the leaf cross-secional area. The sample was then inserted into the preparation chamber (Gatan 1500CT, Gatan, Pleasanton CA USA) of a SEM (JEOL JSM 840A, JEOL, Tokyo, Japan) and evaporatively coated with Al (10 Å) for examination. The initial Pt coat provided an overall superior conduction pathway via the sample substage while the Al-coat on the leaf cross-sectional area provided sample-surface conductivity optimal for x-ray microanalysis. The sample was moved into the SEM specimen chamber and analysed at 15 kV, a take-off angle of 40° and a beam current of 2 x 10^−10^ A. An Aztec analyser with an X-MAX 150 mm^2^ detector (Oxford Instruments, High Wycombe, Buckinghamshire, UK) was used to capture quantitative data and qualitative elemental x-ray analytical maps from selected areas on leaf cross-sectional surfaces. Qualitative maps are shown in terms of counts per second (cps) corrected for background, peak overlaps and pulse pile up events. Quantitative data were obtained by dileanting regions of interest on qualitative maps. The summed x-ray spectra from these regions were processed using the Oxford Instruments version of the XPP software, according to the methodology of Pouchou and Pichoir [43, 44]. Qualitative and/or quantitative x-ray data are represented here for the following elements: oxygen (O), carbon (C), Na, Mg, silicon (Si), P, S, chloride (Cl), K, Ca, Mn.

Since the sample preparation strategy adopted here prioritised cell-content retention over anatomical clarity etc., the secondary electron images (SEIs) were anticipated to lack anatomical clarity. Hence for each X-ray map dataset, an oxygen map was included as a proxy for leaf anatomical detail. Cell-wall Ca evident in Ca x-ray maps provided additional anatomical definition in most samples. The embedding resin Spurrs contained high concentrations of Cl; hence all Cl x-ray data were disregarded. Aluminium data were omitted because the sample was Al-coated. Cell K-content was used to gauge whether cell lumina had been properly retained since translocation of light elements is a common artefact of sample preparation [34, 45]. Consistency of elemental distributions within cellular and subcellular compartments in replicate SEM EDS datasets can be regarded as additional evidence of sample preparation that has effectively retained tissue cell-contents *in vivo*, since artefactual dislocation of cell contents via ion diffusion across cell-wall and cell-compartment boundaries yields anomalous inconsistent x-ray analytical data lacking sharp distributional boundaries. Here, individual x-ray datasets, each acquired over 6–12 hrs, from 2–4 regions within a leaf cross-section, for two different leaf cross-sections (leaves) per tree, from all 8 trees sampled for leaf chemical analysis by ICP, i.e 4–8 x -ray datasets per tree were obtained. Single representations of qualitative pictorial x-ray map data will be included in the Results section below even though they were obtained in multiples. Note, as detailed earlier in this section, leaves sampled and processed for SEM EDS x-ray analysis were not from the pooled leaf material (10 leaves per tree) analysed by ICP. Logistics of sample preparation and the need for a substantial sample size for bulk foliar elemental analysis prevented the division of single leaves for both ICP and SEM EDS analyses. The ICP data in this study were intended as indicators of mean foliar nutrient concentrations per tree, by way of demonstrating agreement with existing literature documenting differences in foliar Mn and other nutrient concentrations between sugar maple and red maple growing on acidified and non-acidified soils of the Allegheny Plateau.

## Results

Leaf chemical analyses by ICP obtained here confirmed existing knowledge about the nutritional status of sugar maple and red maple trees at this and other sites on the Allegheny Plateau, i.e. that both tree species on unbuffered acidified soils upslope accumulate much higher foliar-Mn concentrations than they do on buffered soils downslope (Table 1). Upslope sugar maple had the highest observed foliar Mn concentrations, approximately 5–20-fold that of downslope trees; with around a 2–6-fold difference for red maple (Table 1). Upslope sugar maple foliar-Mg concentrations were around half that of downslope trees. Sugar maple foliar-Al concentrations were greater than those of red maple across slope positions, with both species exhibiting stronger accumulation on acidic soil.

**Table 1:** Foliar elemental concentrations in dry weight (DWT) sugar maple (SM) and red maple (RM) leaves sampled at upslope (U) and downslope (D) positions.

Tree no—Maple species/slope	Dry weight foliar elemental concentrations (mg kg^-1^_DWT_)											
	B	Na	Mg	Al	P	S	K	Ca	Mn	Cu	Fe	Zn
1-SM/U	64	< 10	700	39	2400	1800	6200	7400	4300	9	57	17
2-SM/U	66	< 10	700	41	1500	1500	8600	8600	3200	6	90	22
3-SM/D	36	< 10	1500	25	1300	1700	7100	9100	760	5	42	17
4-SM/D	25	< 10	1100	18	1400	1500	4600	9800	210	6	38	11
5-RM/U	42	< 10	2400	11	1500	1200	6100	8900	2600	9	38	23
6-RM/U	48	< 10	1800	17	1600	1200	6500	7900	1200	11	66	37
7-RM/D	33	< 10	1300	8	1400	1100	6700	6100	390	5	34	21
8-RM/D	32	< 10	1600	8	1300	1100	6200	6500	730	3	48	32

Comparison of quantitative leaf-damage data between slope locations (Table 2) revealed that sugar maple trees on unbuffered acidified soils upslope were more damaged by biotic stress than those on buffered soils downslope (*P < 0*.*02*). While leaf surface damage overall was greatest in the red maple trees, it was similar at both slope locations. Therefore, red maple biotic stress leaf-damage was independent of slope location (*P = 0*.*26*).

**Table 2:** Mean leaf adaxial surface disruption for duplicate sugar maple (SM) and red maple (RM) trees sampled at upslope (U) and downslope (D) positions.

Maple species/slope	n leaves	Mean* ± sd*
SM/U	83	7.74 ± 1.91
SM/D	88	4.57 ± 1.97
RM/U	71	9.54 ± 1.20
RM/D	121	7.95 ± 1.63

* Calculated as reverse arcsin-transformed data

**Fig 1:**
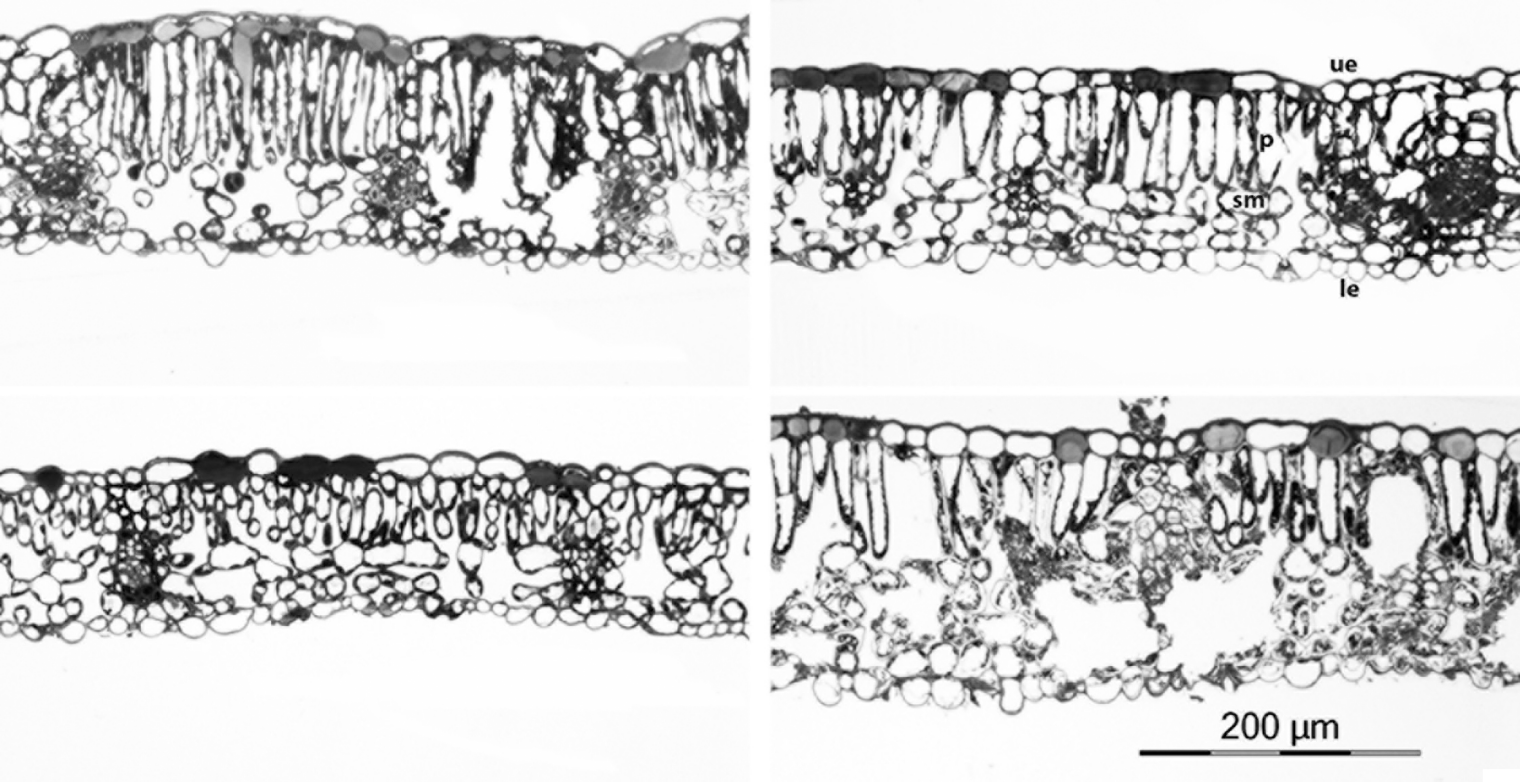
Light micrographs of upslope (above) and downslope (below) sugar (LHS) and red (RHS) maple leaf cross-sections. Upper epidermal (ue), palisade (p), spongy mesophyll (sm) and lower epidermal (le) labels and scale bar apply to all images.

Light microscopical examination of leaf anatomy (Fig 1) showed some natural variation, although this was not attributable to species or tree position. Anatomical features common to all samples included mostly single or less frequently two layers of elongate palisade cells below the upper epidermis and preceding the spongy mesophyll layer set above the lower epidermis. There were intercellular spaces in the spongy tissue and among palisade cells.

**Fig 2:**
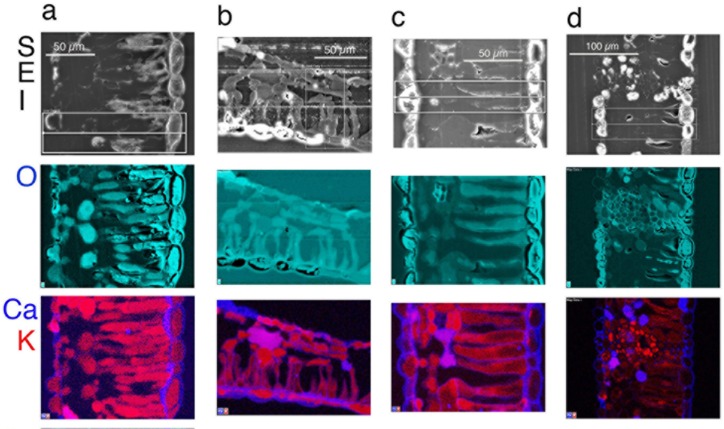
Secondary electron images (SEI) in the top horizontal panel, with corresponding colour-coded X-ray maps directly below, showing *in vivo* O and Ca-K maps in the two panels below. Leaf cross sections were taken from the following trees: (a) sugar maple uplsope (SM/U), tree no.1 in Table 1; (b) sugar maple downslope (SM/D), tree no. 3 in Table 1; c) red maple upslope (RM/U), tree no. 5 in Table 1; and d) red maple downslope (RM/D), tree no. 8 in Table 1. Upper epidermis at RHS in (a), (c) and (d), below in (b).

**Fig 3:**
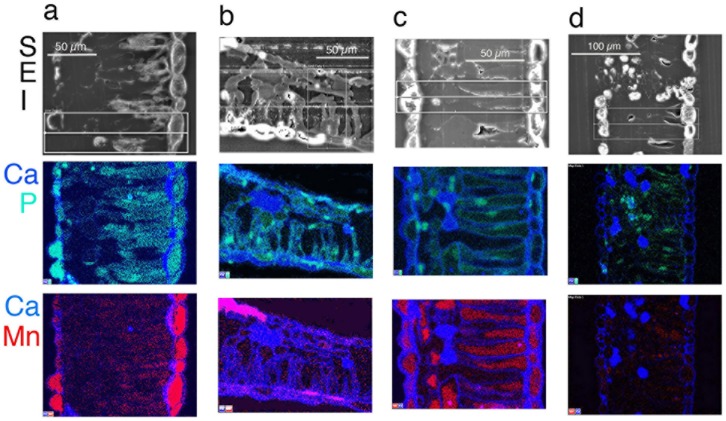
Secondary electron images (SEI) in the top horizontal panel (same sample as in Fig 2), with corresponding colour-coded X-ray maps directly below, showing *in vivo* Ca-P and Ca-Mn composite maps in the two panels below.

**Fig 4:**
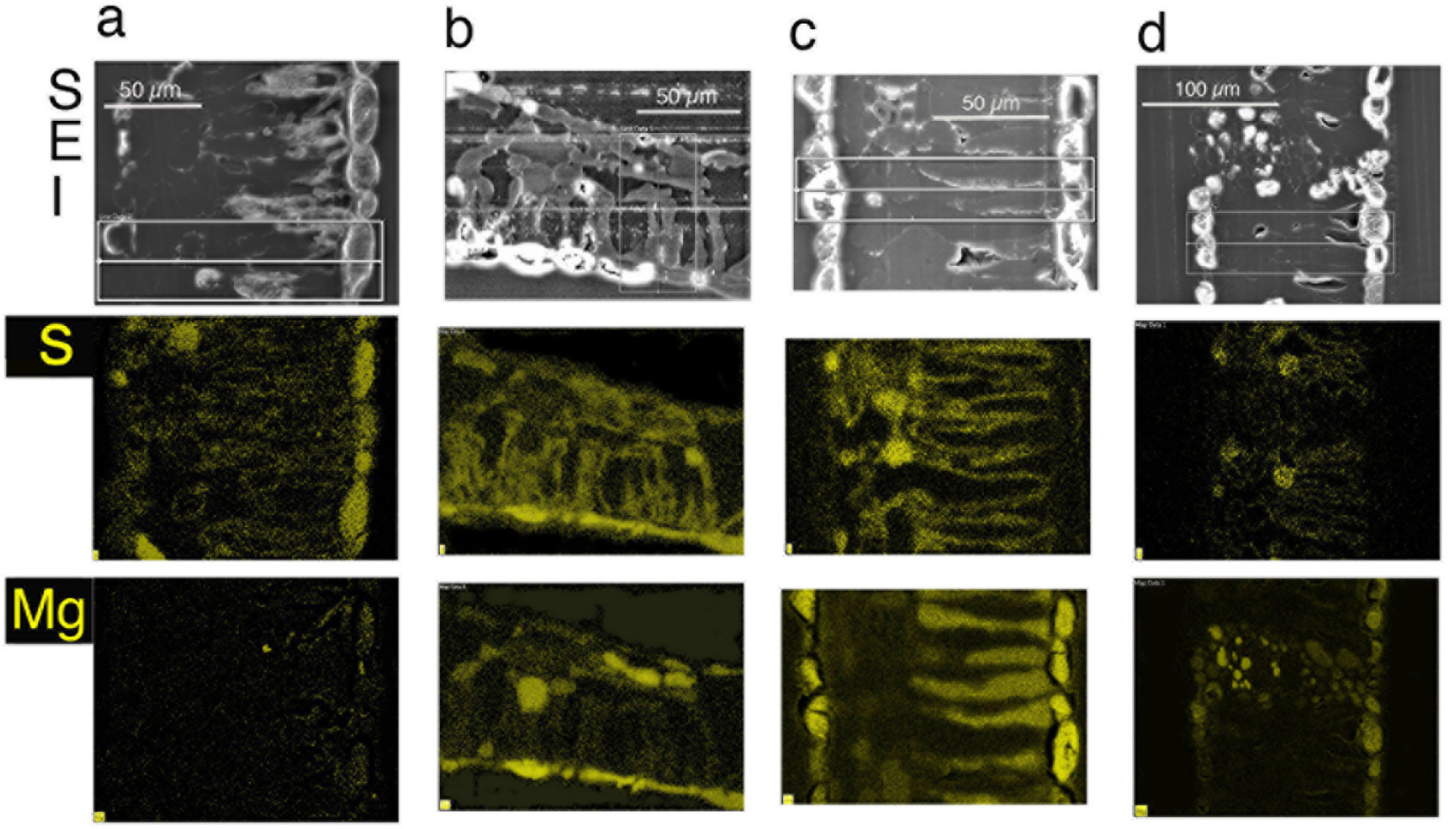
Secondary electron images (SEI) in the top horizontal panel (same sample as in Figs 2 and 3), with corresponding colour-coded X-ray maps directly below, showin *in vivo* S, and Mg maps in the two panels below.

**Fig 5:**
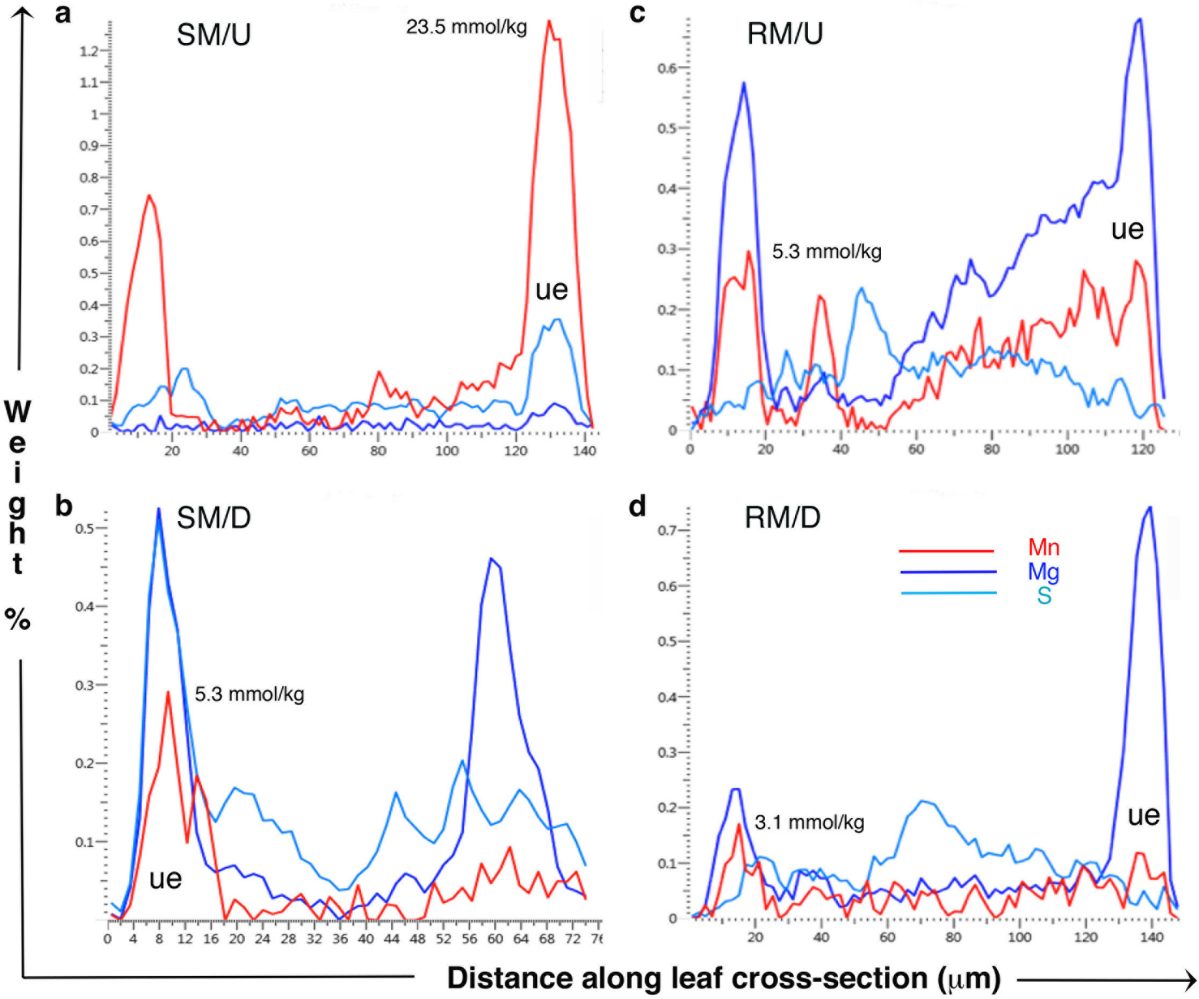
X-ray intensity profiles for Mn, Mg and S along transect lines taken through leaf cross sections as depicted in the SEM images corresponding to frames a, b, c, and d in Figs 1–4. Data are collected to the lateral boundaries marked on either side of the central line. For the largest Mn peak in each frame, weight % Mn concentrations have been converted to mmol/kg as a guide. Notation: ue = upper epidermis.

**Fig 6:**
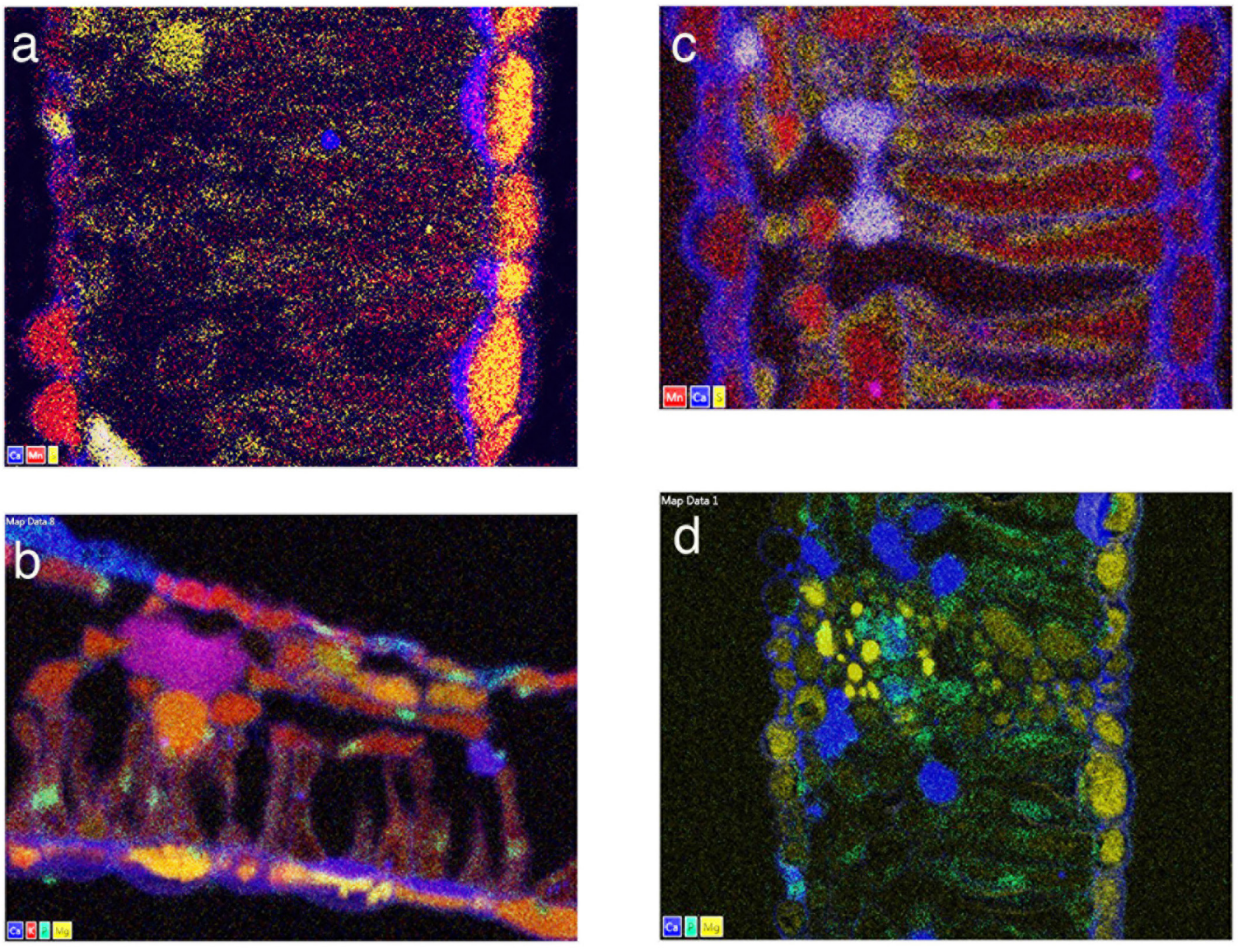
X-ray mapping data from Figs 2–4 combined here to highlight differences in multi-elemental co-deposition patterns in: a) SM/U, b) SM/D, c) RM/U, d) RM/D.

Qualitative and quantitative x-ray data extracted from leaf tissues showed that the *in vivo* micro-distribution patterns of certain nutrient elements such as Mg, S and Mn varied between sugar maple and red maple, and that Ca deposits were least evident in sugar maple upslope (SM/U) (Figs 2–5). These observations were borne out in replicate, of which single representations of elemental x-ray map datasets for sugar maple (Figs 2–4A and 4B) and red maple (Figs 2–4C and 4D) are included here. For any given sample, the intensity of x-ray signal/counts comprising elemental maps (i.e, their clarity) reflected total tissue concentrations of those elements. There was little detectable intraspecific difference between upslope and downslope *in vivo* foliar elemental micro-distribution patterns. Where an element such as Mn occurred in much lower tissue-concentrations downslope for example, it was observed within anatomically equivalent locations at which it was abundant in corresponding upslope samples with highly elevated tissue (Mn) concentrations. This was difficult to resolve when the element was barely detectable. In sugar maple upslope (SM/U), highly elevated foliar-Mn was concentrated in dermal cell vacuoles; however, the sequestration of far lower foliar-Mn concentrations in sugar maple downslope (SM/D) was not clearly resolved, with indication of possible apoplastic Mn deposition in the upper epidermal layer. Vacuolar Mg, S and Mn concentrations extracted from selected areas of Figs 2–4A–4C (Table 3) provide a guide to localised *in vivo* elemental concentrations. X-ray intensity line-scan profiles for Mn, Mg and S collected across leaf sections (Figs 2–4) clearly show interspecies differences in cellular sequestration patterns for these elements (Fig 5). Assembled x-ray mapping data (Fig 6) highlighted key aspects of contrasting foliar elemental sequestration patterns in SM/U, SM/D, RM/U and RM/D samples including the co-accumulation of dermal Mn and S in SM/U only (Fig 6A), dermal Ca-Mg co-deposition in SM/D (Fig 6B), mesophyll and dermal vacuolar-Mn, and mesophyll cytoplasmic S in RM/U (Fig 6C) and vacuolar Mn in dermal and mesophyll cells along with strong Ca deposition in RM/D (Fig 6D).

**Table 3:** Mean vacuolar Mg, S and Mn concentrations in upslope sugar maple (SM/U) and upslope red maple (RM/U) leaves.

Maple species/slope	Leaf cell vacuolar elemental concentrations (mmoles/kg of embedded tissue)						
	cell type (n)	Mg	se	S	se	Mn	se
SM/U	ue (6)	20.6	5.0	77.9	12.7	173.0	47.1
SM/U	p (4)	8.2	2.1	21.8	3.1	32.8	6.2
RM/U	ue (5)	230.5	20.2	9.3	1.4	47.4	4.2
RM/U	p (5)	148.1	12.9	18.7	2.8	41.9	8.4
RM/U	sm (7)	70.0	6.2	24.9	3.5	85.6	11.8
RM/U	le (5)	177.0	23.9	9.3	1.4	54.6	12.5

Cell-type notation: upper epidermis (ue), palisade mesophyll (p), spongy mesophyll (sm), lower epidermis (le).

Dermal cell vacuoles were the primary deposition sites for Mg, S and Mn in all leaf samples, even when their sequestration patterns contrasted markedly between species. Co-localisation of these elements in SM/U and RM/U, of which SM/U had depleted foliar Mg and both SM/U and RM/U had highly elevated Mn were clearly different. Most notable was the highly localised Mg and Mn in RM/U mesophyll cell vacuoles. Upper-epidermal cells were generally well delineated by Si in the cell-walls of most samples examined. The cell walls were Ca abundant, which aided interpretation of tissue anatomy since it delineated cells. Oxygen maps indicated the presence of cytoplasmic, vacuolar and cell wall components that contained higher O concentrations than the surrounding resin matrix. These O maps also suggested the occurrence of crystalline Ca-oxalate when they mirrored corresponding high-intensity Ca x-ray maps in shape and signal-intensity of these angular crystals. Presumed Ca-oxalate crystals and Ca-intense deposits were prevalent through leaf tissues of all samples except SM/U. Amorphous non-oxalic Ca deposits occasionally contained embedded spots/patches of O-intensity, suggesting Ca-oxalate crystal formation *in situ* (Fig 2). There was one observation of dermal Ca-oxalate deposition in SM/D directly matching an area of highly localised Mn (Fig 2B). Cellular K was present throughout, indicating satisfactory tissue fixation and elemental retention. Cell nuclei were detectable by intensely bright P ‘spots’ (Fig 2) as confirmed by TEM. RM/U mesophyll cells containing strongly localised vacuolar Mn had highly detectable S in their cytoplasm, with little or no S in dermal cell vacuoles. However, in SM/U where mesophyll vacuoles did not contain highly concentrated Mn, S was present in the cytoplasm of these cells, and, most notably and in contrast to red maple, strongly localised in dermal cell vacuoles.

## Discussion

Consistent with previous studies, here sugar and red maple growing upslope on acidified host substrates (SM/U and RM/U) on the Allegheny Plateau were found to be in a state of nutritional imbalance by late summer. Both SM/U and RM/U had greatly elevated foliar Mn, with SM/U being additionally Mg-deficient. Findings of similar field studies [13, 22] that RM/U (over)accumulates higher foliar-Mn concentrations than SM/U, and that SM/U is affected by Ca-deficiency were not confirmed by data obtained here, most likely due to seasonal variation, although the lack of Ca-intense deposits such as Ca oxalate in SM/U leaf tissues was noteworthy. Dry weight (DW) phytoavailable soil-Mn concentrations at this study site are not known to be high, e.g. 101 mg kg^-1^_DW_ [22] in contrast to certain areas of eastern Australia for example where inherently high phytoavailable soil-Mn concentrations around 5000 μg kg^-1^_DW_ commonly cause crop toxicity [46, 47]. Since Mn uptake by plants is enhanced by temperature and light, differences here in upslope and downslope foliar Mn concentrations while mostly explained by soil acidity may also have been affected by slope-associated climatic variation such as greater exposure to sunshine and warmth upslope compared to sheltered downslope positions [8, 10, 48–50].

Visible leaf damage measurements (Table 2) incorporating insect herbivory, pathogeny and malnutritional effects suggested that soil acidification had a significant exacerbating effect on sugar maple only, notwithstanding widespread leaf necrosis apparent on red maple trees on both slope positions of the study site. These data provide plausible association between nutritional imbalance, elevated Mn, and a diminished defence capacity in sugar maple. They support the findings of a previous study [51] in which leaf chemistry and biochemistry indicated an acid-soil-enhanced vulnerability to insect damage in sugar maple. Interestingly, and in contrast to observations here on maple (Table 2), intrinsic foliar metal overaccumulation in metallophytes is likely beneficial to chemical defence [52]. It is therefore possible that in this present study, extremely high foliar Mn concentrations distributed throughout the leaves of RM/U provided some degree of chemical defence.

The striking difference in *in vivo* foliar-Mn microdistribution patterns of two species as closely related as SM/U and RM/U was interesting given such intra-generic variation in metal disposal for a single metal is unusual, at least among Mn-metallophytes [33, 53, 54]. Microprobe studies have rarely been effectively applied to Mn accumulation in crop cultivars, some of which while taxonomically almost identical are differentially Mn-tolerant. Cell-fractionation experiments on contrasting Mn tolerance in two bean cultivars have shown foliar Mn to be predominantly vacuolar, i.e. symplastic [55]. Manganese tolerance strongly corresponded to its (vacuolar) sequestration in the dermal fraction, a detoxification strategy likely to minimise damage to vital metabolic processes within the mesophyll. Here, excess foliar-Mn in upslope trees of both maple species were found to be located in dermal cell vacuoles, however concentration within mesophyll cell vacuoles of the less Mn-sensitive RM/U is a novel strategy previously only observed among Mn-hyperaccumulating plant species [56], a small group with an intrinsic ability to scavenge and accumulate extremely high foliar Mn concentrations [57, 58]. These observations alone do not explain the known difference in Mn-sensitivity between SM/U and RM/U [13, 23], a), and should be viewed collectively with other results here of certain specific differences in foliar elemental distributions. Limitations of the methodology precluded detection of very low Mn concentrations, for example as would occur in the photosynthetic apparatus. If Mn present in relatively low foliar concentrations in SM/D was sequestered at least partly within the dermal apoplastic tissues as suggested by the x-ray data, then at greatly elevated foliar Mn concentrations, i.e. in SM/U, this sequestration strategy had shifted to a predominantly symplastic one.

It is reasonable to assume that vacuolar Mn and Mg exist *in planta* in their soluble forms, i.e. as very similar divalent ions Mn(II) and Mg(II) [1, 59, 60]. Mesophyll cells are far more photosynthetically active than dermal cells, which in these maple species appeared to contain almost no chloroplasts when examined by both light microscopy and TEM. Mesophyll Mg(II)/Mn(II) ratios in SM/U likely limit photosynthetic efficiency by excess Mn(II) competing with the Mg(II) Rubisco activase co-factor [61]. It is also known that overaccumulated foliar Mn(II) can cause oxidative stress [9, 13, 22]. Therefore, in Mg-sufficient Mn-accumulative RM/U where foliar Mg is abundantly co-localised with Mn in the mesophyll cell vacuoles, Mg may mitigate oxidative stress by overcoming Mn competition, whereas in Mg-deficient and Mn-sensitive SM/U, excess Mn when overloaded into the mesophyll competes more directly with antioxidative enzyme co-factors to cause stress. The finding here that excess foliar Mn is entirely vacuolar in SM/U affected by Mn toxicity adds to a mounting body of evidence that disagrees with the hypothesis that Mn phytotoxicity occurs in the apoplast, manifesting in dark leaf speckles [4, 6, 31, 62]. Numerous field and controlled-environment studies of Mn toxicity in maple and bean [2, 3, 9, 13, 22, 55] thus far overwhelmingly support the hypothesis that its mediation is symplastic. In these studies, the exacerbating effects of sunlight on Mn toxicity further re-enforces the symplastic nature of these processes. While it is inarguable that some plants upon exposure to excess Mn produce dark leaf-speckles, this may not be strictly a phytotoxcic response, rather, one of overexposure and foliar overaccumulation. It is noteworthy that these studies suggesting an apoplastic scenario for Mn toxicity employed light intensities well below that of solar radiation, thereby precluding the photobleaching and oxidative stress effects reported in maple and bean [4, 31].

Although leaf chemical analyses gave little indication of overall Ca deficiency in either species, it is noteworthy that the microanalytcal data indicated a high frequency of Ca-oxalate crystals and unidentified Ca deposits in all samples except SM/U, previously reported as Ca deficient. The formation of crystalline Ca-oxalate as well as its possible role in regulating Ca in plants as discussed in the literature [63, 64] suggests that while there was no direct evidence here of antagonistic Ca-deficiency driven by Mn oversupply at the root-soil interface, the formation of Ca-oxalate crystals may have been affected. The single observation of Mn co-deposition with dermal Ca-oxalate in RM/D is evidence for a possible Mn-oxalate association, and has previously reported in common bean [55].

In interrogating plant nutritional imbalance at the leaf cellular level, this study has provided new perspective on the impacts of soil acidification and further confirmed that the mediation of Mn toxicity and tolerance is predominantly symplastic. In doing so it helps resolve conflicting theories of the mechanisms of Mn toxicity in the literature. Inherent plant genetic differences in competition, uptake and compartmentation are likely key determinants of susceptibility to nutritional stress, even among species as closely related as sugar and red maple. Plant Mn toxicity in both cultivated and natural systems should be of current and future concern given Mn is common in soil, and easily rendered phytotoxic by climatic changes in process. Given that sugar maple stress continues to be widely documented on soils with relatively low bioavailable Mn concentrations long after early soil acidification should be indicative of the far-reaching impacts of anthropogenically altered climate variables. Other factors such as rising temperatures and warmer summers with extended periods of sunshine are predicted to further exacerbate and extend Mn phytotoxicity. This study highlights the complexities of predicting and elucidating nutritional stress in the face of shifting plant-soil relationships brought upon by climate change.

## Conclusions

Major interspecies differences observed here in the cellular deposition of foliar Mn and Mg suggested that for sugar maple, elevated symplastic Mn in combination with depleted Mg likely hinders photosynthetic efficiency and free radical damage mitigation via competition with enzyme co-factors; whereas in red maple, excess symplastic Mg mitigates Mn toxicity mechanisms in the symplast. Manganese abiotic stress due to Mn overaccumulation in sugar maple foliage exacerbates biotic stress leaf-damage including insect attack.

## Supporting Information

S1 Table Herbarium vouchers lodged at the PAC Herbarium, The Pennsylvania State University. (DOCX)
