# Supplementary material for: Foliar Nutrient Distribution Patterns in Sympatric Maple Species Reflect Contrasting Sensitivity to Excess Manganese
Source: PLoS One. 2016 Jul 8;11(7):e0157702. doi: 10.1371/journal.pone.0157702 (PMC4938512; doi:10.1371/journal.pone.0157702)
Supplement: S1 Table — (DOCX) [file pone.0157702.s001.docx]

**S1 Table:** Herbarium vouchers lodged at the PAC Herbarium, The Pennsylvania State University.

| Species | Voucher number | Slope location  (Hardwood Ridge) |
| --- | --- | --- |
| *Acer saccharum* | PAC106530 | Upslope |
| *Acer saccharum* | PAC 106531 | Upslope |
| *Acer rubrum* | PAC 106528 | Upslope |
| *Acer rubrum* | PAC 106529 | Upslope |
| *Acer saccharum* | PAC 106256 | Downslope |
| *Acer saccharum* | PAC 106527 | Downslope |
| *Acer rubrum* | PAC 106556 | Downslope |
| *Acer rubrum* | PAC 106533 | Downslope |
